# Fostering research integrity in sub-Saharan Africa: challenges, opportunities, and recommendations

**DOI:** 10.11604/pamj.2022.43.182.37804

**Published:** 2022-12-07

**Authors:** Luchuo Engelbert Bain, Larissa Ange Tchuisseu-Kwangoua, Oluwafemi Adeagbo, Ngwayu Claude Nkfusai, Hubert Amu, Farrukh Ishaque Saah, Francis Kombe

**Affiliations:** 1 Department of Psychology, Faculty of Humanities, University of Johannesburg, Johannesburg, South Africa,; 2 International Development Research Centre (IDRC), Ottawa, Canada,; 3 Faculty of Medicine, University of Southampton, Southampton, United Kingdom,; 4 Research Department, Medical Mind Association, Yaoundé, Cameroon,; 5 Department of Community and Behavioral Health, College of Public Health, University of lowa, lowa, United States,; 6 Department of Sociology, University of Johannesburg, Auckland Park, Johannesburg, South Africa,; 7 Malaria Consortium, Yaoundé, Cameroon,; 8 Department of Population and Behavioural Sciences, Fred N. Binka School of Public Health, University of Health and Allied Sciences, Hohoe, Ghana,; 9 Department of Epidemiology and Biostatistics, Fred N. Binka School of Public Health, University of Health and Allied Sciences, Hohoe, Ghana,; 10 African Research Integrity Network, Durban, South Africa,; 11 EthiXPERT, Pretoria, South Africa,; 12 University of KwaZulu Natal, KwaZulu Natal, South Africa

**Keywords:** Research misconduct, ethics, integrity, sub-Saharan Africa

## Abstract

Integrity and adherence to appropriate ethical standards are important elements of research. These standards are key to protecting research participants´ rights as well as ensuring the reliability and quality of research outputs. Although empirical evidence is scanty, several authors have alluded to the fact that violation of research integrity standards could be common in low- and middle-income countries including sub-Saharan Africa (SSA). Understanding the issues, challenges, and opportunities of research integrity and ethics in SSA is key to promoting the responsible conduct of research and the protection of research participants. This paper presents the authors´ critical views and recommendations on the current state of research integrity in SSA. We argue that understanding the current research integrity architecture in SSA has the potential to identify opportunities to promote responsible conduct of research in SSA. Such opportunities include, but are not limited to transparency, accountability, and reproducibility of research, which collectively lead to enhanced public trust in the research enterprise. We highlight the need to embrace equity, fairness, diversity, and inclusivity in the research cycle from conception (priority setting), funding, implementation, dissemination of findings, and scale up. We move on to provide a rationale for understanding the differences and similarities between research ethics and research integrity. Governments, research, and academic institutions must develop multifaceted approaches to promote compliance with principles of research integrity by developing and implementing clear research integrity policies and guidelines that foster responsible conduct of research and prioritize capacity building and empowerment of early career researchers, students, and other targeted key stakeholders.

## Essay

### 1. Introduction

Conducting research with integrity is a critical part of trustworthiness in global health research. However, cases of dubious or detrimental research practices, otherwise known as ‘questionable research practices’ (QRP) and research misconduct have increasingly become common. Research misconduct means fabrication, falsification, or plagiarism in proposing, performing, or reviewing research or in reporting research results [1]. Responsible conduct of research corresponds to conducting research in ways that fulfil the professional responsibilities of researchers, is no doubt every research institution´s endeavour [2,3]. Research integrity and misconduct have evolved over the years. The first case that sparked a buzz among institutions and the scientific community was the “mouse painting research fraud” [2]. In 1974, William Summerlin, a dermatologist at the Memorial Sloan-Kettering Cancer Centre in New York, tried to show that conserving tissues in laboratory culture could avoid transplant rejection in genetically different animals. Nevertheless, a technician found that the white mouse's black posterior stain could be wiped off with ethanol. About a month later, Summerlin admitted that he painted the mice [3,4]. Along with this case, the Vijay Soman and Mark Spector cases caught the eyes of the US congress, which published guidelines and laws regarding research integrity [3]. In May 1992, the Office of Research Integrity (ORI) was created from the fusion of the Office of Scientific Integrity and the Office of Scientific Integrity Review (OSIR) in the Office of the Assistant Secretary for Health (OASH), but these actions were limited to the US [5-7].

Globally, a project of World Conferences on Research Integrity (WCRI), has evolved linking the ORI conference program to the European Science Foundation in Europe, with the first one in Lisbon, in 2007 [8]. Similarly, in 2010, in Singapore, researchers sought to foster research integrity by establishing a guide that could serve as a framework for creating laws, policies, and regulations in different contexts. Four principles namely, honesty, accountability, professionalism, and stewardship, and fourteen responsibilities for the responsible conduct of research were established at the end of the conference. These responsibilities included integrity, adherence to regulations, research methods, research records, research findings, authorship, publication acknowledgement, peer review, conflict of interest, public communication, reporting irresponsible research practices, responding to irresponsible research practices, research environments and societal considerations [9].

Afterwards, several research integrity (RI) conferences has been held offering new insights and updates to approve common principles, create a “Registry for Research on the Responsible Conduct of Research”, and promote financial support for research on research integrity- and formulating principles as the Hong Kong principles among others [10-15]. Overall, there is a global effort to promote research integrity and prevent questionable research practices and research misconduct. Despite the increased global efforts to promote research integrity, little has been done to assess and understand the current situation in sub-Saharan Africa (SSA), including the challenges and opportunities for promoting research integrity as well as African-focused recommendations. This manuscript aims to fill this gap.

### 2. State of the art in SSA

There have been several instances of research misconduct in SSA. Amidst the many research scandals in Africa, some Nigerian studies have shown significant levels of research misconduct among researchers in Nigeria [16,17]. A descriptive study reported over 68% of researchers admitted to having committed at least one form of scientific misconduct, including authorship conflict (36.4%), plagiarism (9.4%) and data falsification (42%), intentional violation of participant recruitment procedures and pressure from sponsors (19.4%) [17]. In addition to this, unethical authorship was the main misconduct reported in a study investigating research collaboration in universities in Nigeria [18]. In Kenya, a survey administered to HIV researchers found that 68% of the investigators in their Kenyan study were involved in at least one misconduct including falsification, plagiarism, selective elimination of outliers, and authorship disputes [19]. Furthermore, the researchers did not obtain ethical approval from one of the two ethics bodies thereby creating loopholes in participants´ rights protection [19].

Further, a case of research misconduct was reported by Weiss *et al*. in which a clinical trial in South Africa claimed to have treated breast cancer using high-dose chemotherapy and subsequently by bone marrow transplantation was not reproducible by other researchers [20]. Another study of research misconduct in South African universities showed falsification and plagiarism as major issues [21]. This prevalence of research misconduct underscores the need to address the research integrity and ethics contexts in SSA. Table 1 shows the summary of findings of some studies investigating research integrity/misconduct in SSA [16,17,19,22-24].

**Table 1:** studies on research integrity/misconduct in sub-Saharan Africa

Author, year	Country	Study design	Population	Summary of findings
Adeleye OA, Adebamowo CA, 2012	Nigeria	Cross-sectional	132 medical/dental researchers	Some 22.0% admitted to at least one of fabrication, falsification, and plagiarism, the predictors of which were knowledge gaps in research ethics and pressure to publish enough papers for promotion. Acknowledging inadequate knowledge of research ethics was a predictor of admitting a wrongdoing
Okonta P, Rossouw T, 2012	Nigeria	Descriptive	133 researchers	It was found that 68.9% admitted having committed at least one of the eight listed forms of scientific misconduct. Disagreement about authorship was the most common form of misconduct committed (36.4%) while plagiarism was the least (9.2%). About 42% of researchers had committed falsification of data or plagiarism. Analysis of specific acts of misconduct showed that committing plagiarism was inversely associated with years in research; falsifying data was related to perceived low effectiveness of the institution’s rules and procedures for reducing scientific misconduct; and succumbing to pressure from study sponsor to engage in unethical practice was related to sex of researcher
Okonta IP, Rossouw T, 2014	Nigeria	Cross-sectional	133 researchers	Half of the respondents (50.4%) were aware of a colleague who had committed misconduct, defined as ‘non-adherence to rules, regulations, guidelines, and commonly accepted professional codes or norms’. Over 88% of the researchers were concerned about the perceived amount of misconduct prevalent in their institution and 96.2% believed that one or more forms of scientific misconduct had occurred in their workplace. More than half (52.7%) rated the severity of penalties for scientific misconduct in their work environment as low. Furthermore, the majority (56.1%) were of the view that the chance of getting caught for scientific misconduct in their work environment was low. Researchers in Nigeria perceive that scientific misconduct is commonplace in their institutions, but are however worried about the negative effects of scientific misconduct on the credibility of scientific research
Were E, Kaguiri E, Kiplagat J, 2020	Kenya	Cross-sectional	100 researchers	53.9% reporting awareness of an incident of RM in the preceding 5 years. Awareness was associated with being in academia, perception of vulnerability to being caught, and the severity of possible punishment, if discovered. Two-thirds (68.3%) reported ever-involvement in any misconduct. Self-report of involvement in misconduct was associated with knowledge of rules and procedures on RM and a disposition to support such rules and regulations. Nearly 36% reported ever-involvement in fabrication, falsification and/or plagiarism (FFP). Self-report of ever-involvement in FFP was associated with number of years in the academic position, perceived likelihood of being caught, and the perceived severity of the sanctions, if caught
Were E, Kiplagat J, Kaguiri E, Ayikukwei R, Naanyu V, 2022	Kenya	Qualitative	27 research regulators	There was no dedicated capacity to prevent or manage research misconduct at the institutional and national levels. The national research regulator had no specific guidelines on research misconduct. At the institutional level, the only capacity / efforts mentioned were directed at reducing, detecting and managing student plagiarism. There was no direct mention of capacity to manage fabrication and falsification or misconduct by non-student researchers
Rohwer A, Young T, Wager E, Garner P, 2017	Low-and-middle countries (LMICS)	Cross-sectional	Corresponding authors of Cochrane systematic reviews working in LMICs	LMIC researchers report that guest authorship is widely accepted and common. While respondents report that plagiarism and undeclared conflicts of interest are unacceptable in practice, they appear common. Determinants of poor practice relate to academic status and power, fueled by institutional norms and culture
Kingori P, Gerrets R, 2016	Sub-Saharan Africa (SSA)	Qualitative study	Fieldworkers of 2 SSA biomedical research institutions	Fabrications were motivated by irreconcilable moral concerns, faltering morale resulting from poor management, and inadequate institutional support. To fieldworkers, data fabrication constituted a ‘tool’ for managing their quotidian challenges. Fabrications ranged from active to passive acts, to subvert, resist and readdress tensions deriving from employment inequalities and challenging socio-economic conditions

Research misconduct is defined as fabrication, falsification, or plagiarism in proposing, performing, or reviewing research, or in reporting research results. Proponents of this view argue that misconduct must be committed intentionally, and the allegation must be proven by sufficient evidence [25]. Plagiarism, on the other hand, is “the appropriation of another person's ideas, processes, results, or words without giving appropriate credit” [25]. In most SSA countries, senior and junior researchers acknowledge its practice among colleagues. Translating from one language to another language was a recurrent feature as well as committing plagiarism and self-plagiarism are common [23].

Fabrication and falsification distort the accuracy of the study and undermine confidence in the investigator. Fabrication is making up data or results and recording or reporting them while falsification is manipulating research materials, equipment, or processes, or changing or omitting data or results such that the research is not accurately represented in the research record [25]. Some researchers in low-and-middle countries (LMICs) usually know a person in their institution who make up or change data [23,24]. In a Nigerian study, researchers admitted that they falsified data [17].

Besides publication-related misconduct, gift or ghost authorship is also seldom [22]. It is a common occurrence that the head of the department or another researcher who has not contributed to the work is added as an author on a paper [23]. This situation triggers a spirit of rebellion among some researchers and does not encourage integrity [26]. Additionally, most institutions lack policies on research integrity, while others have started in South Africa, but have no established procedures to handle emerging cases [26,27]. The impact factor mystery, still highly upheld by funders in making decisions, is a huge disadvantage to African researchers, that feel compelled to publish in such journals to be more competitive in winning grants. This is an area of injustice in global health, that has not received the attention it deserves.

### 3. Challenges and opportunities of research integrity in SSA

#### 3.1. Challenges

##### 3.1.1. Inadequate knowledge and awareness

Even in Western countries where research integrity research and institutionalization are relatively advanced, there are still differences regarding how various stakeholders perceive research misconduct and integrity. In a qualitative study that assessed the attitudes and perceptions of project managers in the biomedical sciences in the industry and academic settings towards research integrity and misconduct issues, there were huge discrepancies [26]. Godecharle and colleagues found different definitions of research misconduct, and consequently different ways of dealing with the same issues [27]. This huge divergence can hamper the much-needed collaboration across fields, disciplines, and stakeholders in upholding the much-needed research integrity standards [28]. As a relatively new discipline in the African continent, much work is needed to map out the state of the art when it comes to the burden and patterns, as well as to empirically investigate actors and pathways through which research integrity and misconduct are propagated. This is the bottom line if setting up any viable and actionable institutional policies to guide practices in the continent are envisaged. The recently launched African Research Integrity Network (ARIN) during the 7^th^ World Conference on Research Integrity, which was hosted by the University of Cape Town in South Africa in May/June 2022, is a huge opportunity to bring together academic and non-academic partners, to reflect on the current research integrity status of the African continent, as well as to drive an Africa-centric and relevant agenda to advance research integrity in the continent [29].

##### 3.1.2. Lack of institutional structures, systems, and guidelines

There is an astute paucity of structures within institutions to deal exclusively with research integrity issues in the African continent [30]. It is plausible that these structures will face similar challenges as research ethics committees (recognition of relevance, and funding for instance). Institutional support, which is a necessary step in making these structures function appropriately is lacking. Furthermore, fair engagement between partners from global North and global South remains wanting. Strategic ignorance displayed by the latter remains an important barrier to fair partnership and collaboration. For instance, collaborative research (helicopter research for instance) remains a reality, with very few authors from Africa occupying first or last positions in African-generated research [31,32]. Beyond this, the slow establishment of oversight structures to verify allegations of plagiarism remains one of the thorns on the side of Africa's burgeoning scientific research community. In a context where laws and regulations for intellectual protection are rare and sometimes absent, this is a major obstacle to the development of a healthy research environment.

##### 3.1.3. Paucity of empirical research on research integrity

There is a dearth of studies assessing the prevalence and distribution of misconduct within African research institutions [17]. Indeed, as we write, there is no authoritative empirical study that has done a robust empirical analysis of research integrity practices, institutional landscape, and actual state of art in SSA. The only way to accurately address risky behaviours, promote responsible behaviours, and clean up the research environment is to conduct such investigations. Such empirical research will also support researcher accountability and the trust of clinicians who rely on the results for their clinical practices.

#### 3.2. Opportunities

##### 3.2.1. Funding for research integrity studies

As funding increases, African scientists will inevitably have to respect ethical principles and integrity. Indeed, many foundations and international funding agencies have provided funding for research in Africa over the past decades, including the Wellcome Trust and the Bill and Melinda Gates Foundation. Africa is an untapped and unknown pool of knowledge from which solutions to many current medical challenges may emerge. Honesty, accountability, professionalism, and stewardship are principles that are promoted and strongly advocated by these institutions.

In parallel, the rise of centres founded by African scientists overseas, such as the African Academy of Sciences (AAS), is generating a mentoring system and stimulating responsible research practices. The multidisciplinary aspect of research is increasingly emerging with the inclusion of other professions, governments, and funding agencies. Further, with the advent of globalization, transnational research, and the possibility of carrying out the same studies elsewhere, African academics will need to align themselves with international benchmarks, for their work to be replicated elsewhere. Global North partners will have to pay attention to academic papers published in African journals. These journals will in turn need institutional and financial support from partners, to improve upon their research integrity and peer review policies to command international respect. Fair and equitable partnerships with institutions from the continent in this direction will certainly be highly welcome. African-based journals, which must be valorised first by African researchers, still have a lot of homework to do in instituting and stating clear research integrity practices in the interest of potential authors.

##### 3.2.2. Diversity, inclusivity, fairness, and equity (DIFE)

For the first time in its history, the World Conference on Research Integrity took place in Cape Town in May/June 2022. Among the key outputs of the conference, is the Cape Town statement-which provides a set of sanctity principles and values to foster fairness, equity, and diversity in global health research, with a special focus on the LMICs context. The uniqueness of the Cape Town statement is premised on its focus on macro-level/systemic inequalities and unfair research practices that provide a fertile breeding environment for irresponsible research practices. The authors of the statement explicitly acknowledged that LMICs face unique and diverse research issues, which can no longer be ignored, and that promoting macro level fairness, equity and diversity can foster engagement between institutions, trustworthiness, mutual respect, diversity and inclusivity which are critical in promoting research integrity [32,33]. Importantly, the statement provides a unique opportunity for contextualisation of research integrity within the LMICs context, and for scholars, academics, and other key stakeholders to actively engage and contribute to shaping the research integrity discourse.

##### 3.2.3. Equity in global health research

Despite the existence of the Montreal Statement on research integrity [10], involving a set of responsibilities for cross-border research, global South researchers have continued to experience limited and unfair competitive funding, authorship and research leadership opportunities, compared to their counterparts from the global North. To date, cases of senior researchers being relegated to fieldworkers´/data collectors, missing out on the list of authors, and lacking decision-making responsibilities in collaborative research involving partners from the global North and South are not uncommon. Ethics dumping, parachute/helicopter research practices and reports of funding opportunities being attributed to the condition that have preserved incentives for questionable research practices are common in present-day global health research. In addition, researchers from the global South are generally underrepresented in development research [32,33]. There is therefore a need for funders, publishers, and institutions, from across the globe to develop policies and guidelines to eliminate these practices. The recent announcements by the Nature and Lancet journals to reject papers that do not include enough local or regional experts from where the research was conducted is one little step in the right direction [34]. This war will however not be won by one single institution or stakeholder. Donors, research institutions, publishers/journals as well as researchers, scholars, and other stakeholders, must add their voices and make their little but invaluable contributions to ensure global research takes place in a fair and equitable environment. Table 2 presents a summary of the challenges and opportunities of research integrity in SSA and recommendations for improving research integrity in SSA [2,9,35-43].

**Table 2:** challenges, opportunities and recommendations for better research integrity in sub-Saharan Africa

Challenges of research integrity in SSA	Opportunities of research integrity in SSA	Recommendations to improve research integrity in SSA
Inadequate knowledge and awareness	Funding for research integrity studies	Education
Lack of institutional structures, systems, and guidelines	Diversity, inclusivity, fairness, and equity	Institutional support
Paucity of empirical research on research integrity	Equity in global health research	Fair and equitable North-South partnerships

SSA: sub-Saharan Africa

### 4. Recommendations to improve research integrity in SSA

#### 4.1. Education

This paper has described the current situation related to research integrity in SSA. One of the key challenges described relates to lack of awareness and understanding of research integrity. We have argued that most research and academic institutions lack systems, procedures and programmes that aim to create or enhance awareness and understanding of research integrity. To address this gap, African-based students and researchers must be properly trained on research integrity. Such education programmes must highlight and underscore the differences between research integrity and research ethics and stipulate the roles and responsibilities of the key players. We have annexed a table presenting the differences between research ethics and research integrity/misconduct (Annex 1). We argue that imported theories and models about research integrity are unlikely to work in developing a conceptually relevant understanding of the scope and magnitude of research integrity malpractices in the continent. Empirical research to document the burden of research integrity, as well as key drivers in SSA is critical. The failures attributable to imported generic research ethics models that failed to respond to local norms and needs must be avoided. The patterns and ways through which researchers falsify data are different [32]. Having SSA-driven data will be important to provide context relevant and appropriate answers in preventing, tracking, and dealing with research integrity issues.

#### 4.2. Institutional support

Setting up clear policies and institutions to oversee research integrity and misconduct practices remain important. This requires strong dialogue, and advocacy from stakeholders to push forward and get the buy-in of appropriate structures from senior management. Challenges could range from human resources, financial, commitment, and inertia. Lessons from the establishment of Research Ethics Committees could be relevant here. A careful situational analysis will be highly needed to come up with a model that meets the challenges and resources. Relevant dialogue among academic and non-academic actors (industry, the Africa Centre for Disease Control, The World Health Organization and The African Union) remains an urgent need in this area. The ARIN stands a huge chance to serve as a flag bearer in this direction.

#### 4.3. Fair and equitable North-South partnerships

Fair and equitable partnerships are highly needed to foster research integrity practices in the continent. Helicopter research remains unacceptable, and appropriate proactive research integrity practices are highly needed. Clear frameworks, including the principles of research fairness initiative developed by the Council on Health Research for Development (COHRED), to guide partnerships throughout the research cycle, from engagement to publication are key. Funders and journals must continue to be vigilant on clear policies that allow for African based authors to occupy respected positions in African based research (first and last authored positions), except in very exceptional cases. Funders have a moral duty to integrate clear integrity best practices, and monitor them, when it comes to North-South research collaborations. Articles published in African journals need to be given the same weight to papers from high impact factor journals from the North. Institutional support for African based journals to improve upon their integrity practices and peer review quality could be a good starting point.

### 5. Conclusion

Despite the increasing positioning of SSA as a global hub for research and development, SSA´s research integrity and ethics practices remain opaque and disfranchised from the global research integrity discourse. Literature regarding SSA researchers´ adherence and compliance with research integrity guidelines and protocols is scanty. Although there has been a plethora of opportunities such as increased funding and mentoring system to improve research integrity and ethics among researchers in the sub-region, there still exists a myriad of challenges facing these efforts. With the irresistible movement towards open science, data sharing and digitalisation of research, which makes consumption of research data instantaneously, real time and globalised, promoting research integrity in and for SSA is no longer a continental/regional affair, but a global discourse that demands the proactive involvement of every practitioner and stakeholder interested in research integrity and most importantly, responsible conduct of research. Inevitably, revolutionizing research integrity practices in SSA will require multifaceted, multisectoral, multidimensional and multicultural approaches that must be backed by clear legal, structural and oversight systems, as well as operational procedures and best practices, aimed at nurturing a responsible conduct of research ecosystem in SSA. While awareness creation and training of researchers, students, and other stakeholders, coupled with the establishment of institutional system to investigate and manage research misconduct may address some of the challenges outline above, we argue that there is no one-size-fit all strategy for addressing this situation. Efforts to co-share, co-create and continued reflection must therefore continue, to allow research practitioners and oversight institutions from SSA to engage in this global discourse and ensure practices of researchers from SSA are open for scrutiny and interrogation.

**Ethics declarations:** the manuscript does not concern the study of persons. All the authors consent to the publication of this manuscript.

## Annex

**Annex 1**: differences between research ethics and research integrity/misconduct
